# The impacts of regional transport and meteorological factors on aerosol optical depth over Beijing, 1980–2014

**DOI:** 10.1038/s41598-018-22803-x

**Published:** 2018-03-23

**Authors:** Xingfa Gu, Fangwen Bao, Tianhai Cheng, Hao Chen, Ying Wang, Hong Guo

**Affiliations:** 1 State Key Laboratory of Remote Sensing Science, Institute of Remote Sensing and Digital Earth of Chinese Academy of Sciences, Beijing, 100101 China; 2 University of Chinese Academy of Sciences, Beijing, 100049 China

## Abstract

Understanding the role of different sources that contribute to the aerosol extinction coefficient is an important aspect toward analyzing climate change and regional air quality. In Beijing specifically, the region has suffered severe air quality deterioration over the past three decades, but the magnitude of extraneous contributions to aerosol variation has remained uncertain. Therefore, we estimated trends of contributions to aerosol optical depth (AOD) for Beijing from 1980 to 2014 and built a seasonal regression model to decouple the extraneous contribution from the total emitted using ground-based aerosol and meteorological measurements, extended to the emissions of man-made and natural contribution. The variation of AOD over Beijing was significantly affected by the anthropogenic aerosol emissions, which experienced slight augmentation by 15.3% from 1980 to 2000, rapid inflation by 36.9% from 2000 to 2006, and a gradual decrease by 10.0% from 2006 to 2014. The extraneous contribution from wind and its associated languishing patterns explain the historical increase of regional AOD, which experienced about a 10% enhancement over the three stages. Other meteorological contributions show no significant trends over 35 years, except for the temperature inversion, which despite the weakened hygroscopic growth after 2006, still experiences a significant enhancement.

## Introduction

Severe haze plumes from highly concentrated aerosols significantly degrade atmospheric quality and, consequently, increase human health risks accompanied by the explosive economization and urbanization in developing countries^1–6^. As the capital of China, Beijing has suffered the worst air quality deterioration after 1980^7–10^, associated with escalating levels of sulfate, carbonaceous, and other aerosols that result from growing fuel consumption since the beginning of industrialization^11,12^. Recently, long-term studies of aerosol properties and particulate matter concentration over China have been widely discussed by many scientists^13–15^, and the origin of these variations has attracted extensive attention^16–18^.

Beijing’s high aerosol loading is commonly attributed to anthropogenic activities that are associated with secondary aerosol formation^19,20^ and long-range natural aerosol flux (e.g., sea salt, dust, and natural organic carbon aerosol)^21^. However, environmental contributions cannot be neglected^22–24^. Sun *et al*.^25^ examined the relative humidity and chemical aerosol records for winter in Beijing, learning that all of the aerosol species increase linearly as a function of humidity in dry environments, and the increased rates for most species are reduced at high humidity levels. Cao *et al*.^26^ reported that high aerosol loading in urban areas is commonly attributed to higher temperatures and urban heat island phenomenon. Zhao *et al*.^27^ suggested that the reduction of precipitation in eastern China is correlated to the high concentrations of aerosols over the last 40 years. Zhu *et al*.^28^ and Wu *et al*.^29^ analyzed the relationship between atmospheric circulation and aerosol concentration using a chemical transport model and they confirmed that the convergence of air pollutants from increased aerosol concentrations over eastern China is due to the weakening of the East Asian Summer Monsoon (EASM).

All of these anthropogenic and natural effects, explicitly offered in the studies, are available for rationalizing the variation of historical aerosol physical properties. However, most long-term analyses have focused on the variation of a single variable and did not decouple the interaction between the natural atmospheric environment and local emissions. The magnitude of horizontal atmospheric transport contribution significantly affects the distribution over the region and has remained uncertain. This uncertainty also affects the evaluation of regional aerosol concentrations, making the contribution of the local source and extraneous aerosol flux illusive.

In this study, we present an empirical analysis to show the historical aerosol variation from 1980 to 2014 over Beijing using ground-based aerosol optical depth (AOD) data and meteorological sounding measurements. The impacts of the prevailing wind on aerosols were studied by regression analysis in hopes of providing supplementary evidence and quantitative analysis for transportation conditions related to the autochthonic emission and extraneous transported aerosol pollution. Understanding the geographic sources that contribute to AOD is an important step in furthering aerosol-climate research and generating insight into the explanation of the anthropogenic and natural components.

## Results

### Overall patterns of wind and its effects on AOD over Beijing from 1980 to 2014

**Figure 1:**
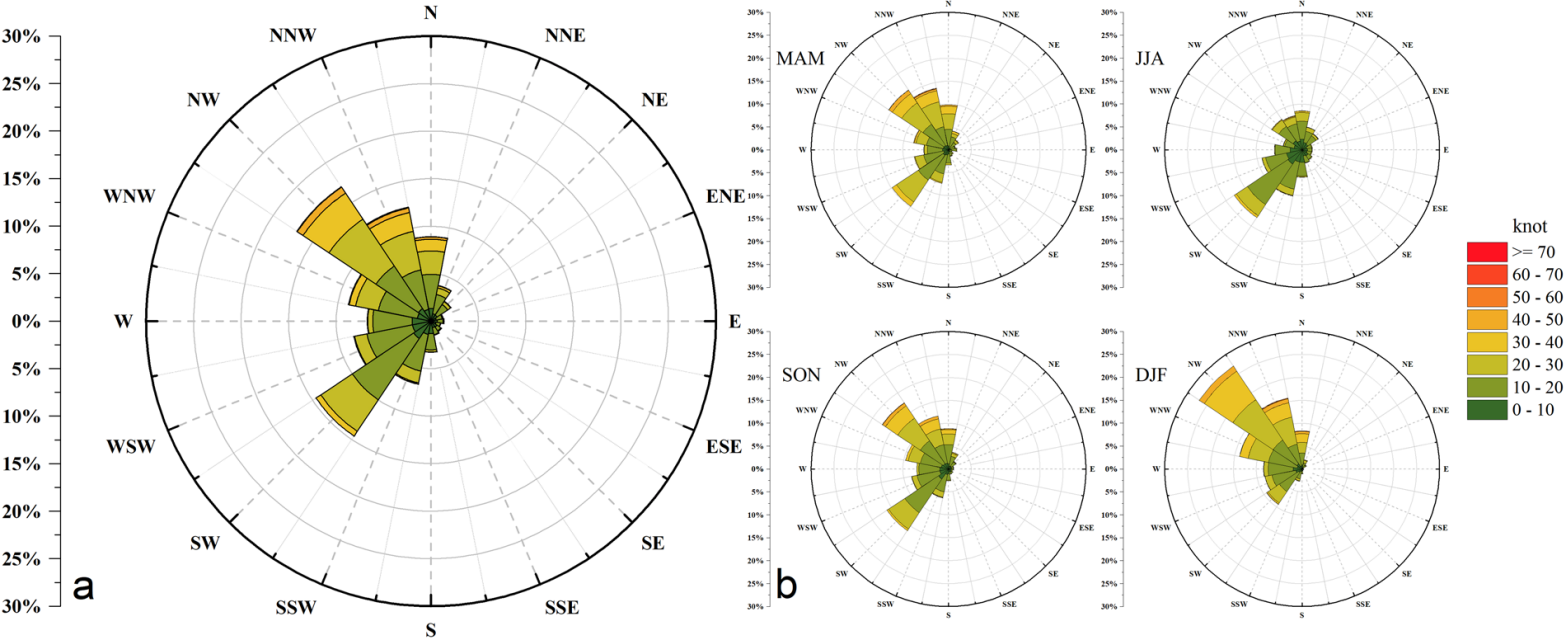
Prevailing wind direction and wind velocity at 850 hPa over Beijing. (**a)** Statistics from 1980–2014, and (**b)** seasonal statistics from 1980 to 2014, separated by Spring (MAM), Summer (JJA), Autumn (SON), and Winter (DJF). The color in the figures represents the different levels of wind velocity (in knots) from various directions.

AOD is the most important physical parameter for characterizing aerosols, and also one of the dominating factors for evaluating regional atmospheric contamination and climatic effect^30,31^. In fact, AOD is different from surface aerosol concentration; however, many researchers have used this extinction coefficient to describe regional pollution^32–36^. It has a positive correlation relationship with aerosol concentration and the correlation varies with the seasons (Fig. S1). This light extinction coefficient can be determined by not only local mass concentration, but also the extinction efficiency and hygroscopicity of aerosols, making the relationship more discrete over the seasons^37^. Moreover, many researchers have demonstrated that high aerosol forcing formed a distinctive distribution feature, along with the typical topography contours, indicating the close correlation between the poor dispersion of aerosol pollution and valley mega relief over Beijing^38,39^. In fact, under this “easy piled up” terrain (Fig. S2), it is not difficult to speculate the impact of regional atmospheric transport on aerosol flux^40^. Figure 1 illustrates the status of the wind at 850 hPa in Beijing over 35 years. Compared to the dominant westerly wind at 850 hPa, the northerly and southerly wind generally shares the same proportion over 35 years. In addition, the wind has significant seasonal variations due to the Coriolis force and EAM^41^. The northerly and southerly wind nearly bisect the proportion in MAM and SON, while the opposite is seen in JJA and DJF. In contrast, the westerly wind had a great proportion in each season.

**Figure 2:**
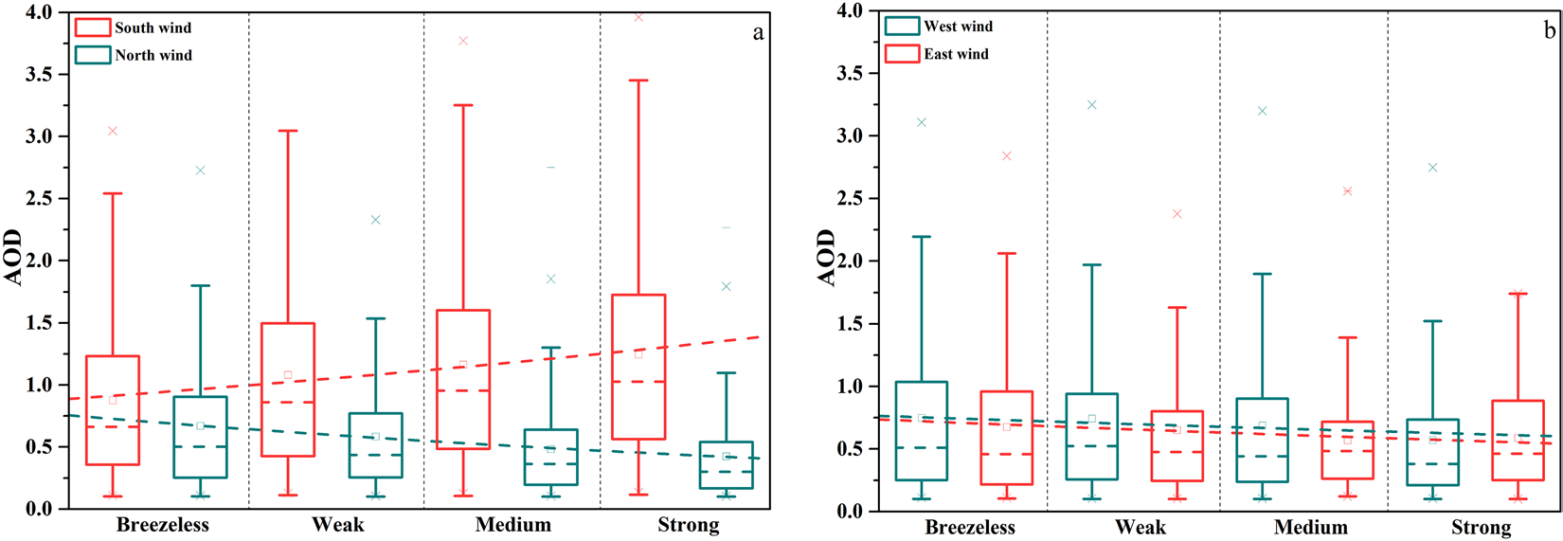
Correlation between AOD and wind properties. (**a)** Variations of the daily AOD correlate with the northerly and southerly wind conditions, and (**b)** easterly and westerly wind conditions, separated by four levels of wind intensity: breezeless (0–5 knots), weak (5–10 knots), medium (10–15 knots), and strong (>15 knots). The dotted line is the exponential fit of the averaged WV - AOD scatter plot.

In fact, the direction of the wind induces different impacts on the AOD. Figure 2 illustrates two different statistical relationships: northerly-southerly and easterly-westerly wind versus daily AOD. It is demonstrated that AOD fluctuations usually occur under different intensities and directions of the wind. The southerly wind easily collects the aerosols over the region, while the northerly wind is beneficial for the aerosol pollutants to diffuse if conditions accelerate from 0 to 20 knots (with a step of 5 knots). However, with the wind acceleration, the impacts of the easterly and westerly wind on the AOD seem equivalent. Furthermore, the regional AOD seems to be only related to velocity, with a slight dispersion effect on the AOD similar to the effect from the northerly wind.

### Separating the autochthonic emission and meteorological contributions on AOD

Separating local emissions and meteorology effects is an essential procedure for analyzing the impacts of various anthropogenic and natural factors of pollution. Actually, it is difficult to decouple these two effects in the trend analysis because the precise description of aerosol composition requires *in situ* chemical measurements that are always restricted in time^42,43^. However, it is possible to estimate the anthropogenic and natural part of aerosols using an aerosol model and chemical model simulation^21,44,45^. In order to discover the potential interaction between the AOD and surface aerosol particle concentration, some researchers separate the meteorological contribution from the total aerosol concentration using meteorological parameters and regression analysis^36,46,47^. Planetary boundary layer height (HPBL), relative humidity (RH), temperature inversion (TI), and wind velocity (WV) are the considerable meteorological parameters in these regression studies. Even though regression models may depend on different meteorological predictors, they all use the same linear functions to define the natural contribution to particle concentration^36,48^.

Based on the previous studies of Liu *et al*.^36^ and Zang *et al*.^47^, we applied an optimal regression model to define the relationship between the windy AOD and the breezeless AOD using several meteorological parameters. Since the wind is an important carrier of extraneous aerosol particles, the geographic sources of aerosols can be easily distinguished by wind intensity, which is defined as follows:

1 $$\{\begin{array}{rcl}{\tau }_{B} & = & f({\tau }_{a})\times f({\tau }_{n})\,(|WV| < 5\,knots)\\ {\tau }_{W} & = & f({\tau }_{a})\times f{({\tau }_{n})}^{\text{'}}\times f(WV)\,(|WV| > 5\,knots)\end{array},$$

where ${\tau }_{B}$ and ${\tau }_{W}$ represent the AOD under the breezeless and windy environments, respectively; $f({\tau }_{a})$ represents the autochthonic contribution on the AOD; $f({\tau }_{n})$ represents the meteorological environmental contribution on the AOD; and $f(WV)$ represents the extraneous aerosol contribution delivered by the wind. $WV$ is a vector parameter for a function of wind speed and wind direction, representing the northerly-southerly or easterly-westerly wind velocity at the maximum of 850 hPa.

Then, equation (1) can be converted into the lognormal optimal regression model by detailing the specific meteorological parameters (further detailed in the Methods section):

2 $$\begin{array}{rcl}\mathrm{ln}\,\tau  & = & {\gamma }_{0}\,\mathrm{ln}({\tau }_{B})-{\gamma }_{1}\,\mathrm{ln}(HPBL)-{\gamma }_{2}\,\mathrm{ln}(TI\_D)-{\gamma }_{3}\,\mathrm{ln}(TI\_T)-{\gamma }_{4}\,\mathrm{ln}(f(RH))\\  &  & +\,{\beta }_{1}\,\mathrm{ln}(HPBL^{\prime} )+{\beta }_{2}\,\mathrm{ln}(TI\_D\text{'})+{\beta }_{3}\,\mathrm{ln}(TI\_T\text{'})+{\beta }_{4}\,\mathrm{ln}(f(RH^{\prime} ))\,\\  &  & +\,{\beta }_{5}W{V}_{NS}+{\beta }_{6}W{V}_{EW},\end{array}$$

where *γ*_0_ − ${\gamma }_{4}$, *β*_1_ − *β*_5_ are the regression coefficients associated with the meteorological parameters under breezeless conditions ($HPBL$, $TI\_D$, $TI\_T$, $f(RH$)) and windy conditions ($HPBL\text{'}$, $TI\_D\text{'}$, $TI\_T\text{'}$, $f(RH\text{'})$, $WV$). Two parameters are defined by the intensity of temperature inversion: $TI\_D$ represents the depth of the inversion layer; $TI\_T$ represents the temperature difference of the inversion layer;$\,W{V}_{NS}$ and $W{V}_{EW}$ represent the wind velocity under the northerly-southerly and easterly-westerly wind, respectively; and $f(RH$) represents the hygroscopic influencing factor, which is expressed in many studies^34,49,50^ as:

3 $$f(RH)=\frac{1}{1-RH}.\,$$

All of the coefficients can be calculated by the abundant values via the Ordinary Least Squares (OLS) method. The meteorological predictors have obvious seasonal variations that do not always significantly contribute to the seasonal AOD variation. In order to figure out the primary contribution of meteorological predictors in each season, the confidence coefficients (CC) were calculated for all of the predictors (Table 1). Apart from the contribution of local aerosol emissions, it is notable that the aerosol flux driven by the northerly-southerly wind is a significant meteorological predictor (CC > 90% or P < 0.1) for all of the seasons. On the contrary, even if the easterly-westerly wind shows slight dispersion effects on the AOD in Fig. 2(b), the dispersions over the seasons seem insignificant on the AOD in the regression study, with the exception of the northerly-southerly wind. In addition, other meteorological predictors, including $HPBL$, $RH$, and autochthonic $TI\_T$ and $TI\_D,$ show a strong dependence on the seasons. The aerosol properties over Beijing were also found to depend highly on the season, the total AOD maxima in the MAM and JJA and minima in the SON and DJF^51,52^. These discrepancies between seasons are mainly attributed to the significant meteorological predictors that cause peaks in the AOD, due to the higher planetary boundary layer height^53–56^, dominant dust aerosol types originating from northern arid regions and deserts in spring (MAM)^57–61^, and the enhanced hygroscopic growth of aerosol in summer (JJA)^48,62–64^, respectively. Therefore, the effects of the significant predictors described in the regression study should also be analyzed in the long-term research.

**Table 1:** Seasonal confidence coefficient (P-value) of all of the meteorological predictors in regression analysis.

	γ_0_	γ_1_	γ_2_	γ_3_	γ_4_	${{\boldsymbol{\beta }}}_{1}$	${{\boldsymbol{\beta }}}_{2}$	${{\boldsymbol{\beta }}}_{3}$	${{\boldsymbol{\beta }}}_{4}$	${{\boldsymbol{\beta }}}_{5}$	${{\boldsymbol{\beta }}}_{6}$
MAM	**0.001**	**0.090**	0.876	0.929	0.312	**0.092**	0.593	0.143	0.182	**4.260E-05**	**0.905**
JJA	**9.84E-06**	0.660	0.882	0.381	**0.062**	0.412	0.299	0.608	**0.058**	**0.002**	**0.684**
SON	**0.009**	0.401	0.754	**0.062**	**0.026**	0.511	0.710	**0.048**	**0.039**	**8.800E-05**	**0.720**
DJF	**0.007**	0.143	**0.011**	0.522	0.184	0.525	**0.089**	0.539	0.870	**3.770E-08**	**0.859**

Table 2 shows the estimated regression coefficients for the significant predictors in the four seasons. Insignificant predictors were neglected from our study due to their low impact on aerosols. Additionally, the northerly wind has strong dispersion effects on aerosols, but the southerly wind brings more pollutants from southern emission sources. These two contributions of the wind are not same and should be opposite in sign. Therefore, in our study, we separated the regression coefficients of the northerly-southerly wind (${\beta }_{5})$ into two parts to reflect the different contributions of the northerly wind versus the southerly wind. Due to the high correlation coefficient (R^2^) in each regression equation, the contribution of the significant natural impacts and autochthonic emissions can be evaluated using these coefficients. Interestingly, all of the predictors, except for wind velocity, are greater than 0, indicating that the meteorological predictors suggested a positive relationship in improving the AOD. On the contrary, two regression coefficients of *WV* are negative, proving that the horizontal atmospheric transportation shows a diluted capability on aerosol concentration under the northerly wind (*WV* > 0, ${e}_{\,}^{{\beta }_{5-N}WV} < 1$), but agminated ability under the southerly wind (*WV* < 0, ${e}_{\,}^{{\beta }_{5-S}WV\,} > \,1$).

**Table 2:** Seasonal estimated regression coefficients using the monthly samples collected from 1980 to 2014.

	γ_0_	γ_1_	γ_2_	γ_3_	γ_4_	β_1_	β_2_	β_3_	β_4_	β_5-N_	β_5-S_	R^2^	RMSE
MAM	0.182***	0.141*				0.121*				−0.028***	−0.019***	0.767	0.167
JJA	0.379***				0.340*				1.097*	−0.022***	−0.018***	0.661	0.239
SON	0.204***			0.027*	0.321**			0.104*	0.054*	−0.039***	−0.025***	0.811	0.116
DJF	0.172***		0.025**				0.123**			−0.048***	−0.027***	0.896	0.168

*Significant at the α = 0.1 level. **Significant at the α = 0.01 level. ***Significant at the α = 0.001 level.

### Historical variations of extraneous and autochthonic contributions to the AOD over Beijing

**Figure 3:**
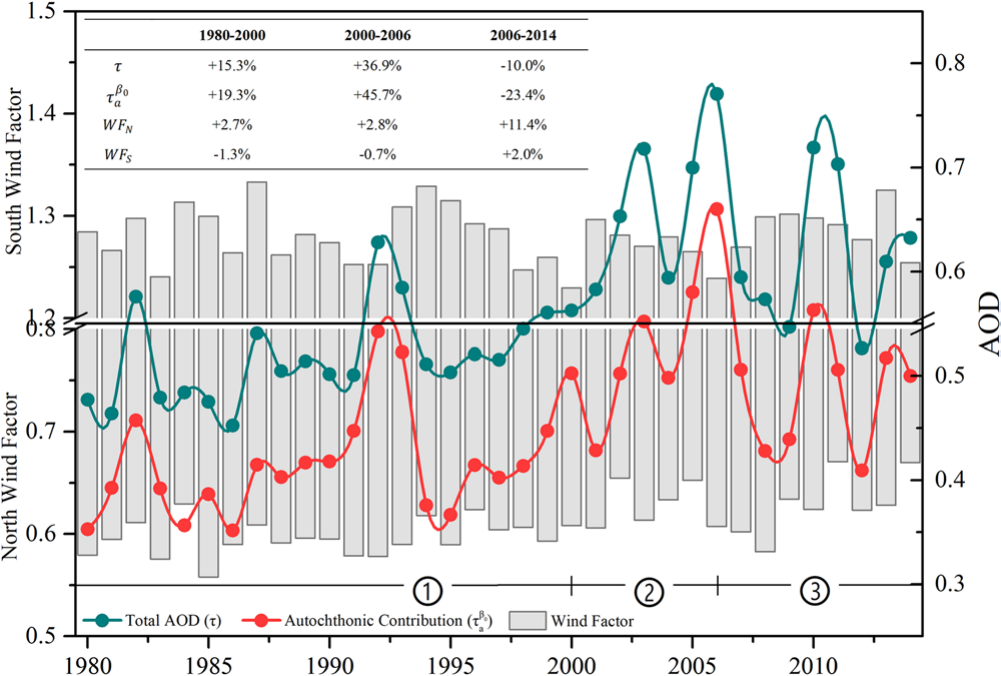
Annual variation of autochthonic emission (${\tau }_{a}^{{\beta }_{0}}$) and extraneous contribution (WF separated by the southerly and northerly wind) on the historical AOD ($\tau$) over Beijing from 1980 to 2014. The table summarizes the trend of three parameters over three stages (1980–2000, 2000–2006, and 2006–2014).

The variation of autochthonic emission (${\tau }_{a}^{{\beta }_{0}}$) and the northerly/southerly extraneous delivered impacts (WF) calculated by regression analyses that are associated with historical AOD ($\tau$) over Beijing from 1980 to 2014 are shown in Fig. 3. The regional AOD experienced a fluctuant increase over 35 years. Accompanied by the different periods of economic and GDP growth of Beijing, the augmentation of total AOD can be divided into three stages: a slow rise from 1980 to 2000 (+15.3%), rapid inflation from 2000 to 2006 (+36.9%), and a gradual reduction from 2006 to 2014 (−10.0%). These significant variations are inseparable from autochthonic emissions and extraneous contributions.

The wind properties over Beijing fluctuated from changing urban structures and weakening of the EAM during the past several decades^65,66^. The weaker northerly wind or stronger southerly wind over the region also influenced the aerosol flux over 35 years, which lessened the diluted capabilities and strengthened the extraneous aerosol contribution. The diluted capability of the northerly wind sees a significant continuous decrease over time, dropping about 2.7% before 2000, decreasing 2.8% from 2000 to 2006, and weakening about 11.4% after 2006. In contrast, the agminated contribution of the southerly wind experienced a stable fluctuation (≤± 2%) over 35 years. As a whole, the aerosol enhancement caused by horizontal wind transports increased about 10% over the 35 years. Moreover, the variation of the autochthonic AOD generated by the local anthropogenic and natural aerosols over the region also saw a remarkable growth over 35 years, with a 19.3% increase before 2000, a 45.7% rapid rise from 2000 to 2006, and a 23.4% decrease after 2006. Since the long-term changes of natural aerosols over East Asia are small, and the slopes of the AOD trends are less than 0.001, the annual changes are less than 1%^21^, so the significant growth of autochthonic emission in our study can be attributed to the explosive anthropogenic emission over the years. The recent remission was attributed to the increasing public concerns of atmospheric pollution and worldwide events, such as the Olympic Games and APEC, which in turn increased government intervention and green energy applications over China. Fig. S3 presents the historical cement or steel production variation, which indicated that the trends of the AOD and autochthonic contribution in our study are consistent with the anthropogenic industrial manufacturers. In addition, Streets *et al*.^21^ noted that the contributions of the major aerosol source types (anthropogenic sulfate) over East Asia experienced about a 46% augmentation from 1980 to 2006 (+51% autochthonic aerosol in our study). Klimont *et al*.^67^ and Lu *et al*.^14^ estimated that the rapidly changing landscape of SO_2_ emissions (main pollutant) in China has resulted in a 30% decrease from 2005 to 2010 (−23% autochthonic aerosol in our study). Boys *et al*.^68^ proposed a view by satellite numerically derived PM2.5 in which the East Asian time series of fine particles steadily increases until 2007, then levels off. These simulated exhaust emissions of inorganic aerosols (SO_2_, CO, PM2.5, etc.) emitted from industrial activities experienced a similar variation over three stages compared to the results of our study.

**Figure 4:**
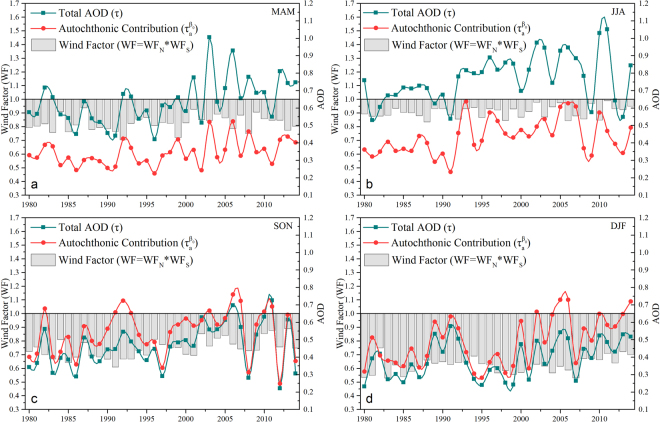
Historical autochthonic emission and extraneous delivered impacts on the AOD over Beijing from 1980 to 2014, separated by season (**a–d**).

Figure 4 illustrates that the seasonal patterns of extraneous factors, autochthonic emissions, and the total AOD experienced a significant change over the last 35 years. The changes of these three parameters over escalating and decreasing periods are summarized in Table 3. The historical AOD rose rapidly before 2006, with a 39.1% rise in MAM, 49.8% rise in JJA, 66.5% rise in SON, and 33.1% rise in DJF. The seasonal autochthonic emissions also experienced a sharp increase before 2006; the regional aerosol emission was enhanced about 28.9%, 73.0%, 42.9%, and 58.5% from spring to winter. The changes of EAM before 2006 slightly weakened the seasonal diluted capabilities of the northerly wind and enhanced the extraneous aerosol contribution, especially in SON, when the northerly wind contribution increased about 19.3%. Additionally, compared to the significant increase before 2006, all three of the parameters showed a certain degree of reverse or continuous deterioration after 2006. The AOD and autochthonic contribution in each season sees a considerable decrease in recent years, except during winter, when coal heating is still active in the city. The increasing demand of burning coal due to an explosive urban population led to a deterioration of aerosol emission (still +35.2% of the AOD, and +26.7% of the autochthonic aerosols). The wind factors experienced a continuous decrease after 2006, especially in winter, when the diluted ability of the northerly wind weakened about 20.7% and the agminated abilities of the southerly wind strengthened about 8.8% on the AOD, creating unfavorable contaminant diffusion conditions over these 9 years.

**Table 3:** AOD (${\boldsymbol{\tau }}$), autochthonic AOD (${{\boldsymbol{\tau }}}_{{\boldsymbol{a}}}^{{{\boldsymbol{\beta }}}_{0}}$), and wind contribution (WF) changes separated by two stages from 1980 to 2014.

	${\boldsymbol{\tau }}$		${{\boldsymbol{\tau }}}_{{\boldsymbol{a}}}^{{{\boldsymbol{\beta }}}_{0}}$		WF (WF_N_/WF_S_)			
	1980–2006	2006–2014	1980–2006	2006–2014	1980–2006		2006–2014	
MAM	+39.1%	−10.4%	+28.9%	−11.1%	+4.6%	+1.1%	−2.7%	+0.3%
JJA	+49.8%	−20.2%	+73.0%	−31.1%	+5.6%	−2.2%	+10.8%	+2.7%
SON	+66.5%	−32.8%	+42.9%	−38.0%	+19.3%	−3.4%	+6.9%	+3.5%
DJF	+33.1%	+35.2%	+58.5%	+26.7%	+1.2%	−1.8%	+20.7%	+8.8%

Furthermore, compared with the wind factors between MAM and SON, which have similar wind structures and properties during the period (Fig. 1), the wind contribution seems smaller in MAM. The main reason is that the northerly wind in MAM is usually interspersed with the coarse aerosol models, so the dominant northern dust flux weakens the diluted capability of the prevailing northerly wind and strengthens the local AOD. In fact, a study of dust-wind interactions has been proposed by Yang *et al*.^69^, and they showed that based on model simulations, the dust emissions decreased by 29% from 1981 to 2015 and they were accompanied by weakened wind speed.

### Impacts of meteorological environment on historical AOD variation over Beijing

**Figure 5:**
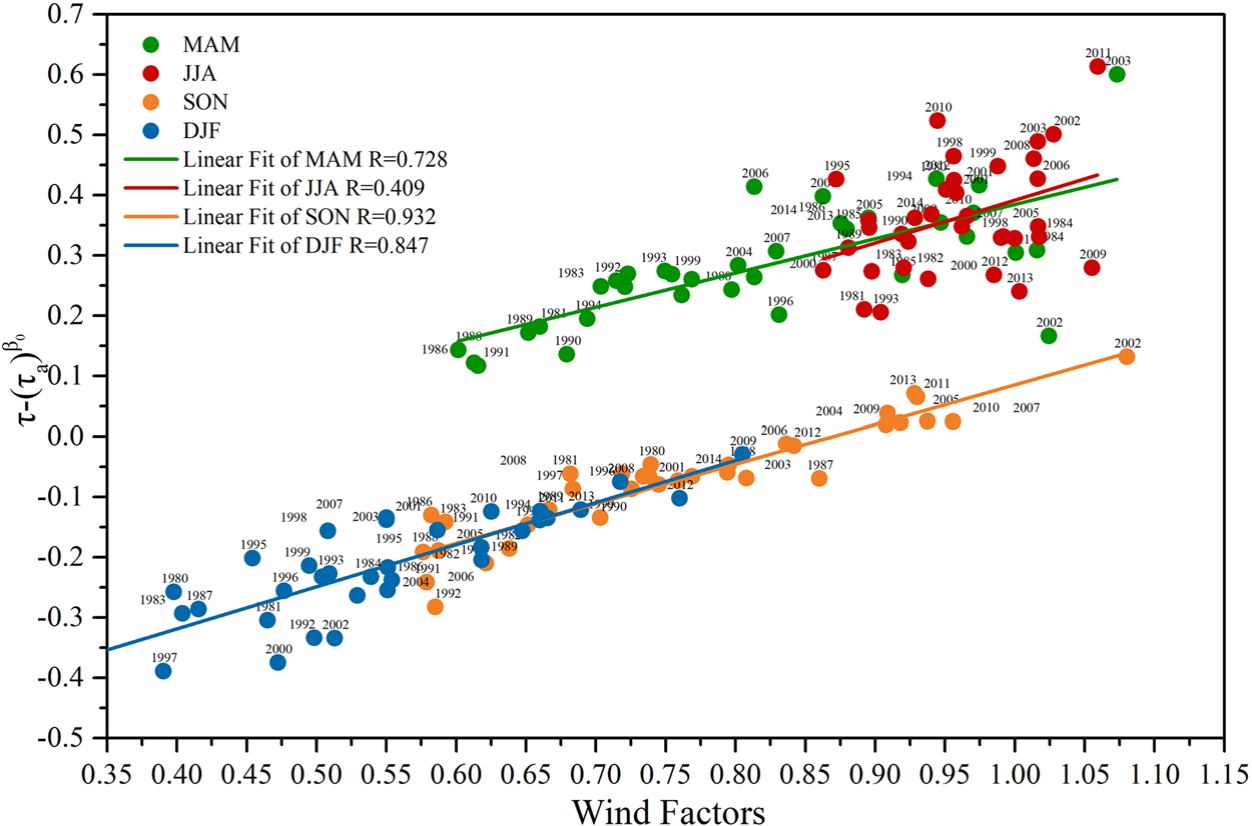
Seasonal correlation between wind factors versus the discrepancy between the AOD (τ) and autochthonic contribution (τ_a_^β0^).

**Figure 6:**
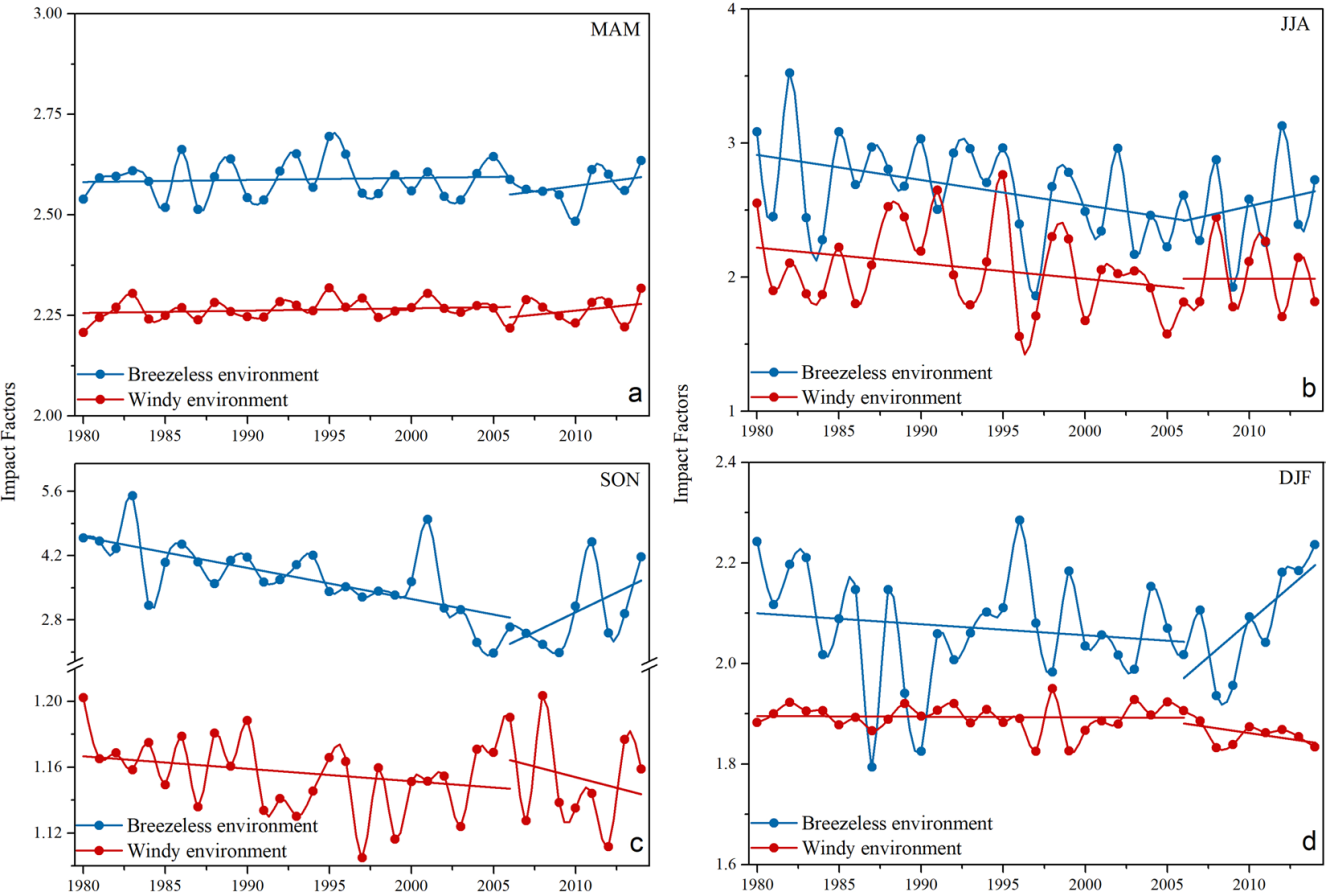
Historical variation of significant meteorological impact factors in each season over Beijing from 1980 to 2014. HPBL in MAM, RH in JJA, TI_T in Son, and TI_D in DJF were included under breezeless and windy environments, respectively. The red lines represent the trend before and after 2006.

Apart from the contribution of autochthonic emission and the wind, some local meteorological parameters also affect the variation of the AOD in history. The significant meteorological impact factors in the seasons can be isolated and evaluated using the estimated regression coefficients in Table 2, but other non-significant meteorological parameters in each season are neglected in our study. In Fig. 4, the fluctuant reduction of the wind contribution in the seasonal analyses is associated with the increasing ‘gap’ between total and autochthonic AOD. The weaker aerosol flux in some years exaggerated the positive effects of other significant meteorological predictors. Figure 5 illustrates the seasonal correlation between the wind factors versus the discrepancy between the AOD and autochthonic AOD. The correlation coefficients (R) are statistically significant in MAM, SON, and DJF (0.728, 0.932, 0.847, respectively), compared to that of JJA (0.409). The higher correlation coefficients also signified strong dependence of the extraneous contribution. Even though the wind counteracts the magnitude of most meteorological impact factors, the historical variation of the significant meteorological factors still has an appreciable impact on the discrepancy between autochthonic emission and the total AOD, especially when the contribution is weak, and becoming a dominant natural factor for escalating AOD. For example, the discrepancy between the AOD and autochthonic contribution in 2009 and 2011 shows a great imparity in Fig. 5, even if they have the same wind factors. According to the historical variation of significant meteorological impact factors in each season over Beijing from 1980 to 2014 (Fig. 6), this imparity was mainly attributed to the significant difference of RH in these years.

The historical variations of the seasonal meteorological predictors are shown in Fig. 6 and the escalating rates over two stages (1980 to 2006 and 2006 to 2014) are summarized in Table 4. Under windy conditions, most meteorological factors did not obviously (<2% in both two stages) change during 3 decadal. However, we could see some significant variation when we designate 2006 as the time node—the hygroscopic growth of aerosol significantly weakened in JJA, accompanied by a drier atmosphere regardless of the windy or breezeless environment. In addition, the synergistic contribution of atmospheric temperature inversion and hygroscopic growth of aerosol in SON was obviously fluctuant over 35 years, with a 39.0% decrease before 2006. Moreover, both the significant meteorological predictors in SON and DJF experienced a recovery after 2006; however, these two augmentations are caused by different reasons. Due to the slight change of breezeless TI_T in SON (Fig. S4), the main reason for the 37.7% inflation after 2006 is the hygroscopic growth of aerosol. The enhancement of the AOD in breezeless DJF resulted from the intensity of the atmospheric temperature inversion, producing a significant augmentation with a 13.7% surge after 2006.

**Table 4:** Changes of significant meteorological predictors under breezeless (MFB) and windy environment (MFW) over two stages (1980 to 2006 and 2006 to 2014).

	MFB		MFW	
	1980–2006	2006–2014	1980–2006	2006–2014
MAM (HPBL)	+0.5%	+1.9%	+0.7%	+1.7%
JJA (RH)	−17.2%	+10.4%	−14.1%	−0.1%
SON (TI_T*RH)	−39.0%	+37.7%	−1.9%	−2.0%
DJF (TI_D)	−3.0%	+13.7%	−0.0%	−0.7%

## Discussion

The regional aerosol concentration can not only be affected by the autochthonic aerosol emission, but also diluted or aggregated by the extraneous aerosol flux and the natural environment. In this study, we focus on the comprehensive quantitative studies about the magnitude of extraneous and meteorological contributions in aerosol variation, attempt to decouple the different sources from the total aerosol properties, and present a long-term seasonal analysis of aerosol variation over Beijing.

In order to isolate the contribution of autochthonic emission and natural factors from seasonal aerosol properties, we applied a lognormal optimal regression model to define the relationship between autochthonic and extraneous aerosol using several meteorological parameters. The confidence coefficients and regression coefficients were calculated for these meteorological predictors and illustrated a strong seasonal dependence. We found that autochthonic emissions and extraneous contributions delivered by the wind are the most significant predictors in the regression model. Otherwise, different local meteorological factors demonstrate dependency in different seasons (HPBL for spring, RH for summer, TI_T and RH for autumn, and TI_D for winter). These predictors have also presented a dominant effect on the regional AOD throughout history.

Using *in situ* measurements and calculated regression results over the region, we found that the AOD changes exponentially, either by increasing or decreasing, and it usually occurs under varying intensities of the northerly or southerly wind. The southerly winds easily aggregate the aerosols over the region while the northerly winds are beneficial for the aerosol pollutants to diffuse when the wind accelerates from breezeless to strong. The seasonal subdued aerosol flux delivered by the wind also contributes to the regional AOD enhancement over the period. In addition, the stronger autochthonic contributions were considered to be a main factor that affected the historical long-term trend of AOD before 2006. After that, both AOD and autochthonic contributions declined gradually due to the increased government intervention and green energy applications over China. The change of wind patterns also influenced the aerosol flux over 35 years; compared to the southerly wind, the continuous weakened diluted capabilities of the northerly wind was the main reason over this time period. Other meteorological contributions showed no such rise or even abated during the same period, which means that the local meteorological environments are not the prime leading factors for the overall annual AOD enhancement over 35 years. However, the meteorological environments, especially the hygroscopic growth of aerosol and temperature inversion impacts, show a significant opposite trend before and after 2006. Even if both significant meteorological predictors in SON and DJF experienced a recovery after 2006, these two augmentations are caused by different factors. The main reason for the inflation in SON after 2006 is the hygroscopic growth of aerosol, while the enhancement of the AOD in breezeless DJF resulted from the intensity of the atmospheric temperature inversion. Overall, the variation of the AOD in Beijing’s history can be separated into three main stages: a slow rise of 15.3% from 1980 to 2000 (mainly caused by +19.3% of autochthonic emission), a 36.9% rapid inflation from 2000 to 2006 (mainly caused by +45.7% of autochthonic emission), and a 10.0% gradual reduction from 2006 to 2014 (mainly caused by −23.4% of autochthonic emission but +11.4% for the weakened extraneous northerly wind).

The results in our study provide a new foundation for identifying the autochthonic emission and meteorological contribution, as well as the roles they play in issues, such as local and regional air quality degradation, and the variation in history. Actually, the autochthonic AOD (${\tau }_{a}$) described in our study still contains two component sources: natural aerosol and anthropogenic aerosol, which are much more difficult to isolate because the coefficients (${\alpha }_{0}$, ${\beta }_{0}$) cannot be evaluated. However, according to Streets *et al*.^21^, the long-term changes in natural emissions over East Asia are small and the least squares slopes of the AOD trends are less than 0.001, indicating that the annual changes of natural emissions are less than 1%. Therefore, the autochthonic contribution proposed in our study is basically identical to the anthropogenic emission variation over 35 years. In our future work, we aim to obtain more precise sounding and aerosol data (hourly scale), broaden the temporal resolution, narrow our focus even more to the other AOD contributors, compare our results with other atmospheric models, and accurately separate the anthropogenic and natural contribution using aerosol chemistry and other meteorological parameters.

## Data and Methods

### Aerosol optical depth data

The AOD is an important parameter for studies of atmospheric pollution, aerosol radiation-climate effects, and remotely sensed atmospheric corrections. Recently, much better estimates of aerosol impacts can be studied over the historical remotely sensed measurements of the AOD, which are directly proportional to meteorological factors. Thus, we can use these measurements of the AOD to evaluate the historical variation of air quality in Beijing. In this study, daily, averaged monthly, and annual AOD at 550 nm were retrieved using the method presented by Luo^13^ from the data of daily direct solar radiation, sunshine duration, ground pressure, and vapor pressure at radiation stations over Beijing from 1980 to 2012. The standard errors of optical depth solutions in the retrieval are all <5% for every atmospheric model. In addition, AErosol RObotic NETwork (AERONET), a federation of ground-based remote sensing aerosol networks established by NASA and PHOTONS, was also used for precision validation and provided absent daily observations from radiation stations. Fig. S5 illustrates the validation study of the daily averaged radiation stations AOD (retrieved) as compared to the AERONET AOD (observed) using simultaneous data from 2002 to 2012. The radiation stations AOD (retrieved) (about 1623 points) exhibits a strong correlation with AERONET, accompanied by a high correlation coefficient (R = 0.880) and desired slope and y-intercept (Y = 0.907 * X + 0.091). Moreover, the average AOD from 2001 to 2012 is 0.443 retrieved from radiation station observations, which is consistent with AERONET (the averaged AOD from 2001 to 2012 is 0.492). Therefore, due to the small discrepancy between the two different datasets, the regional AOD over Beijing can be represented by combining aerosol properties with radiation station and AERONET observations.

### PM2.5 data

Particulates less than 2.5 micrometers in diameter (PM 2.5) are referred to as surface “fine” particulates and are believed to pose the largest health risks. Those effects are likely to be more severe for sensitive populations, including people with heart or lung disease, children, and older adults. The U.S. Embassy provided an air quality monitor to measure PM 2.5 particulates as an indication of air quality above their urban compound (since 2008).

### Radiosonde data

The radiosonde measurements in this study were obtained from the Integrated Global Radiosonde Archive (IGRA) and they consist of radiosonde and pilot balloon observations from over 2,700 globally distributed stations^70^. The measurements of atmospheric pressure, geopotential height, relative humidity, wind (direction and velocity), and vertical profiles of potential temperature were collected at the Beijing (ZBAA) station from 1980 to 2014. In our study, RH and wind properties were collected at 850 hPa. Most researchers who focus on the relationship between general atmospheric circulation and aerosol chose observations at 850 hPa because the atmospheric environment can easily express the real condition of the surface atmosphere and it is not influenced by surface buildings and topography^71–74^. The intensity of temperature inversion was expressed by two factors, the depth of inversion layer (TI_D), and the temperature difference of the inversion layer (TI_T), which can be detected from significant levels because of its unusual temperature lapse profile. The multiple layers were calculated by summing the absolute values of each layer.

### Planetary boundary layer height (HPBL) data

Historic HPBL data were collected from the Twentieth Century Reanalysis Project, which is produced by the Earth System Research Laboratory Physical Sciences Division from NOAA and the University of Colorado Cooperative Institute for Research in Environmental Sciences; they assimilate surface observations of synoptic pressure^75^. The HPBL coincided with the radiosonde station and interpolated from the four closest grids using the bilinear interpolation method. In addition, the aerosols above the HPBL are scarce and contribute little to AOD^47^, thus the temperature inversion that occurred above the HPBL was neglected in our study.

### General description of regression method

In order to decouple the autochthonic and extraneous contribution from the total AOD, we used regression analysis to calculate the contribution of each factor. Here, we classified the autochthonic and extraneous AOD via the threshold of the wind intensity and developed an exponential regression model as follows:

4 $$\{\begin{array}{rcl}{\tau }_{B} & = & f({\tau }_{a})\times f({\tau }_{n})={\tau }_{a}^{{\alpha }_{0}}\times HPB{L}^{{\alpha }_{1}}\times TI\_{D}^{{\alpha }_{2}}\times TI\_{T}^{{\alpha }_{3}}\times f{(RH)}^{{\alpha }_{4}}\,(|WV| < 5)\\ \tau  & = & f({\tau }_{a})\times f({\tau }_{n})^{\prime} \times f(WV)={\tau }_{a}^{{\beta }_{0}}\times HPBL{^{\prime} }^{{\beta }_{1}}\times T{I}_{D}{^{\prime} }^{{\beta }_{2}}\times T{I}_{T}{^{\prime} }^{{\beta }_{3}},\\  &  & \times \,f{(RH)^{\prime} }^{{\beta }_{4}}\times f(WV)(|WV| > 5)\end{array}$$

where

5 $$\{\begin{array}{lll}f(RH)\, & =\, & 1/(1-RH)\\ f(WV) & =\, & \,{e}_{N}^{{\beta }_{5}WV}\times {e}_{S}^{{\beta }_{6}WV}\times {e}_{EW}^{{\beta }_{7}WV}\end{array},\,$$

where ${\tau }_{B}$ represents the AOD under breezeless environment; and $f({\tau }_{a})$ and $f({\tau }_{n})$ represent the autochthonic emission and natural contribution, respectively. Here, we should assume that the autochthonic contribution on the AOD in windy and breezeless periods remains unchanged in a certain scale (monthly in our research). $WV$ represents the wind intensity at 850 hPa and stands for the pure transport contribution of the wind. According to the analysis in Fig. 2, since the AOD contribution discrepancy is opposite in northerly-southerly directions but little in easterly-westerly directions (little depends on intensity), we define the contribution of $WV$ as an exponential form and three parts are separated, including the northerly wind (${e}_{N}^{{\beta }_{5}WV}$), southerly wind (${e}_{S}^{{\beta }_{6}WV}$), and easterly-westerly wind (${e}_{EW}^{{\beta }_{7}WV}$); and ${\alpha }_{0}$ − ${\alpha }_{4}$ and ${\beta }_{0}$ − ${\beta }_{5}$ represent the various regression coefficients for different predictors. Here, different coefficients are proposed in two conditions due to the discrepancies of meteorological parameters under different atmospheric conditions (Table S1). Then, the equation can be converted into the lognormal optimal regression model:

6 $$\{\begin{array}{rcl}\mathrm{ln}\,{\tau }_{B} & = & {\alpha }_{0}\,\mathrm{ln}({\tau }_{a})+{\alpha }_{1}\,\mathrm{ln}(HPBL)+{\alpha }_{2}\,\mathrm{ln}(TI\_D)+{\alpha }_{3}\,\mathrm{ln}(TI\_T)+{\alpha }_{4}\,\mathrm{ln}(f(RH))\\ \mathrm{ln}\,\tau  & = & {\beta }_{0}\,\mathrm{ln}({\tau }_{a})+{\beta }_{1}\,\mathrm{ln}(HPBL^{\prime\prime} )+{\beta }_{2}\,\mathrm{ln}(TI\_D\text{'})+{\beta }_{3}\,\mathrm{ln}(TI\_T^{\prime} )+{\beta }_{4}\,\mathrm{ln}(f(RH^{\prime} ))\\  &  & +\,{\beta }_{5}W{V}_{N}+{\beta }_{6}W{V}_{S}+{\beta }_{7}W{V}_{EW}\,\end{array}.$$

By subtracting the two equations in equation (6), we can eliminate the contribution of autochthonic emission $\mathrm{ln}({\tau }_{a})$, so the regression model can be described as:

7 $$\{\begin{array}{rcl}\mathrm{ln}\,\tau  & = & {\gamma }_{0}\,\mathrm{ln}({\tau }_{B})-{\gamma }_{1}\,\mathrm{ln}(HPBL)-{\gamma }_{2}\,\mathrm{ln}(TI\_D)-{\gamma }_{3}\,\mathrm{ln}(TI\_T)-{\gamma }_{4}\,\mathrm{ln}(f(RH))\\  &  & \,+\,{\beta }_{1}\,\mathrm{ln}(HPBL^{\prime} )+{\beta }_{2}\,\mathrm{ln}(TI\_D\text{'})+{\beta }_{3}\,\mathrm{ln}(TI\_T\text{'})+{\beta }_{4}\,\mathrm{ln}(f(RH^{\prime} ))+{\beta }_{5}W{V}_{N}\\  &  & +\,{\beta }_{6}W{V}_{S}+{\beta }_{7}W{V}_{EW}\,\\ {\gamma }_{i} & = & {\alpha }_{i}{\beta }_{0}/{\alpha }_{0}={\gamma }_{0}\,\ast \,{\alpha }_{i}\,(i=1,2,3,4)\,\end{array}.$$

By applying the abundant observations of the AOD and meteorological parameters, all of the coefficients in equation (7) can be calculated via the OLS method, and the autochthonic emission (${\tau }_{a}^{{\beta }_{0}}$) and each natural environmental contribution can be decoupled after the coefficients are determined.

## Electronic supplementary material

Supplementary Infomation
