# Supplementary material for: The impacts of regional transport and meteorological factors on aerosol optical depth over Beijing, 1980–2014
Source: Sci Rep. 2018 Mar 23;8:5113. doi: 10.1038/s41598-018-22803-x (PMC5865206; doi:10.1038/s41598-018-22803-x)
Supplement: Supplementary file 1 — Supplementary Infomation [file 41598_2018_22803_MOESM1_ESM.docx]

**The impacts of regional transport and meteorological factors on aerosol optical depth over Beijing, 1980-2014**

**Xingfa Gu**^a,†^**, Fangwen Bao**^a,b,†^**, Tianhai Cheng**^a,*^**, Hao Chen**^a^**, Ying Wang**^a^**, and Hong Guo**^a^

aState Key Laboratory of Remote Sensing Science, Institute of Remote Sensing and Digital Earth of Chinese

Academy of Sciences, Beijing 100101, China;

bUniversity of Chinese Academy of Sciences, Beijing 100049, China;

†These authors contributed equally to this work.

*To whom correspondence should be addressed. Email: chength@radi.ac.cn


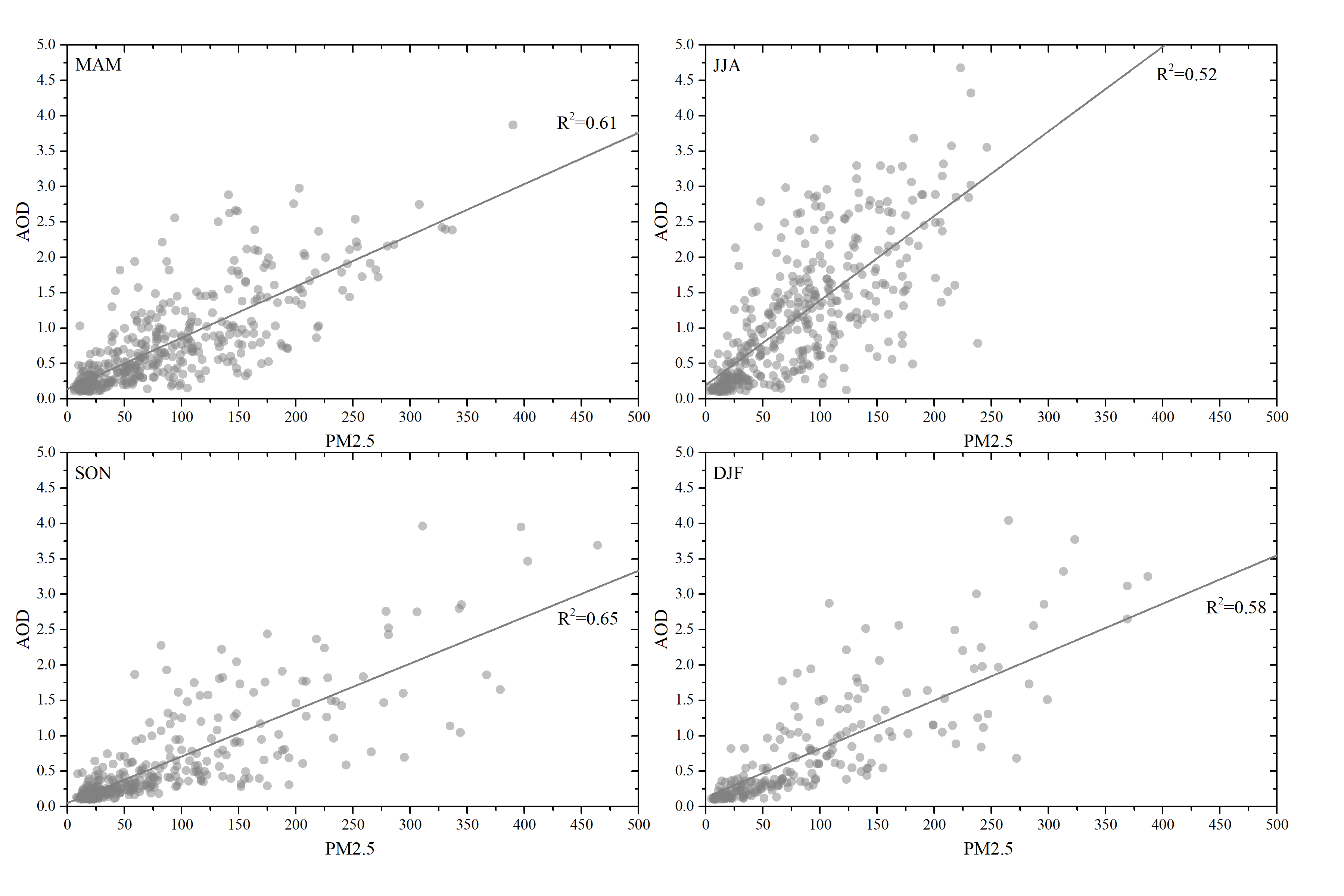


**Supplemental Figure S1 | Seasonal ground level PM 2.5 over the U.S. Embassy of Beijing as function of sun photometer AOD from 2008 to 2014.**


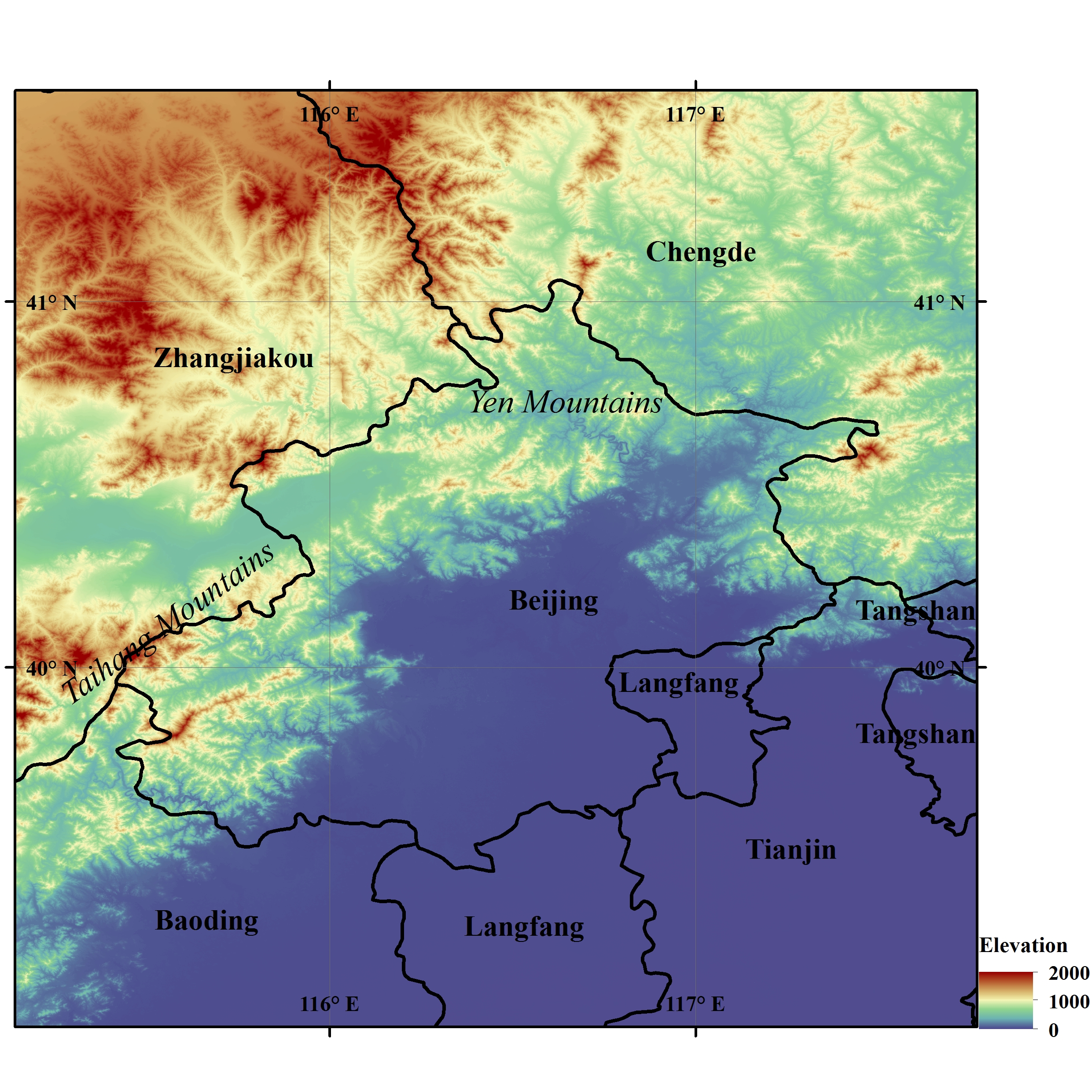


**Supplemental Figure S2 | The location and topography of Beijing generates by ARCGIS using Advanced Spaceborne Thermal Emission and Reflection Radiometer Global Digital Elevation Model (ASTER GDEM) data.** The presented ASTER Global Digital Elevation Model (GDEM) data raster map is a product of NASA and METI and is distributed by NASA LP DAAC (<https://lpdaac.usgs.gov/>). ArcGIS 10.3 software (http://www.esri.com/software/arcgis) was used to develop the map.


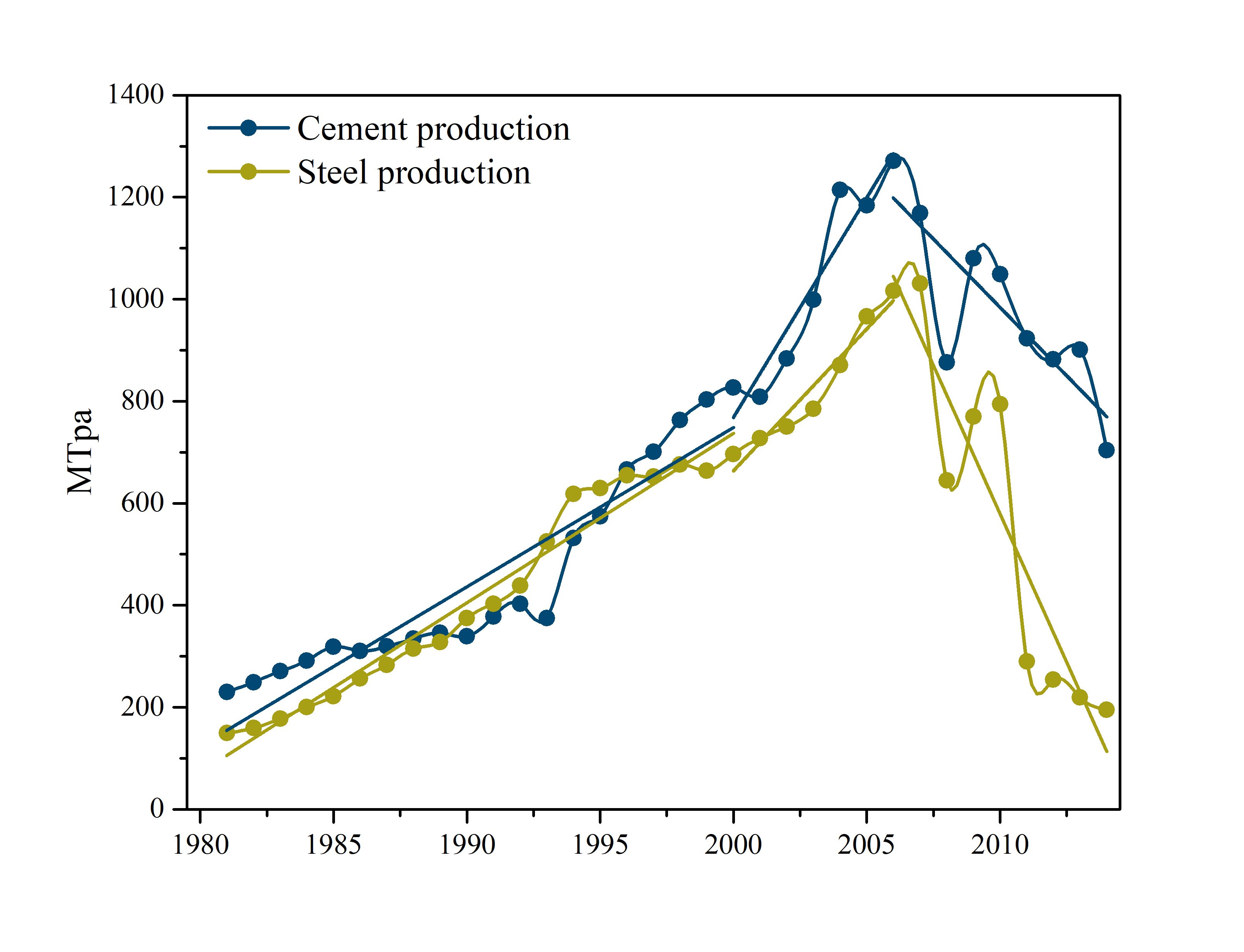


**Supplemental Figure S3 | Historical variation of cement production and steel production over Beijing collected from** **National Statistics Bureau of China.**


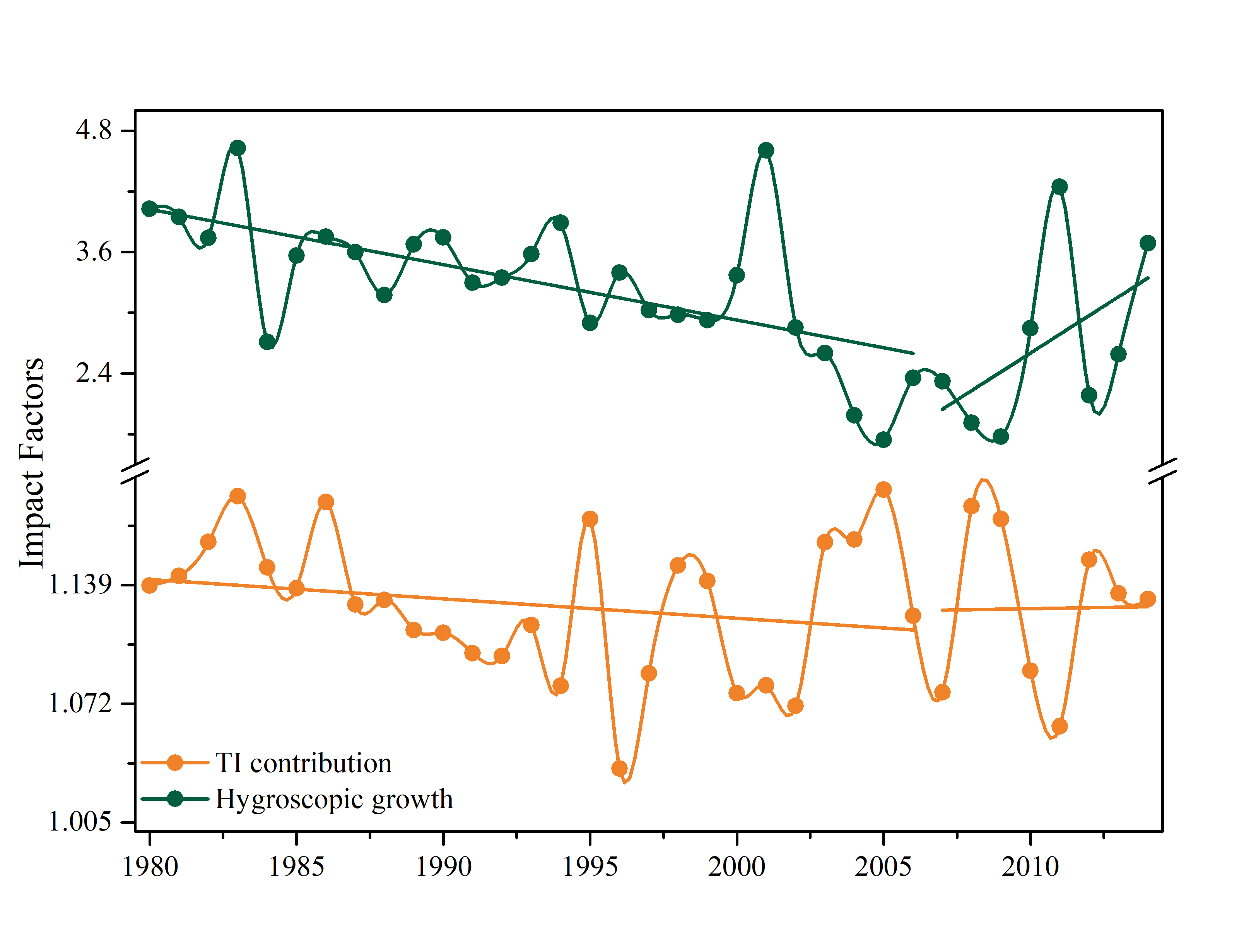


**Supplemental Figure S4 | Historical variation of TI contribution and Hygroscopic growth in autumn (SON) over Beijing from 1980 to 2014.**


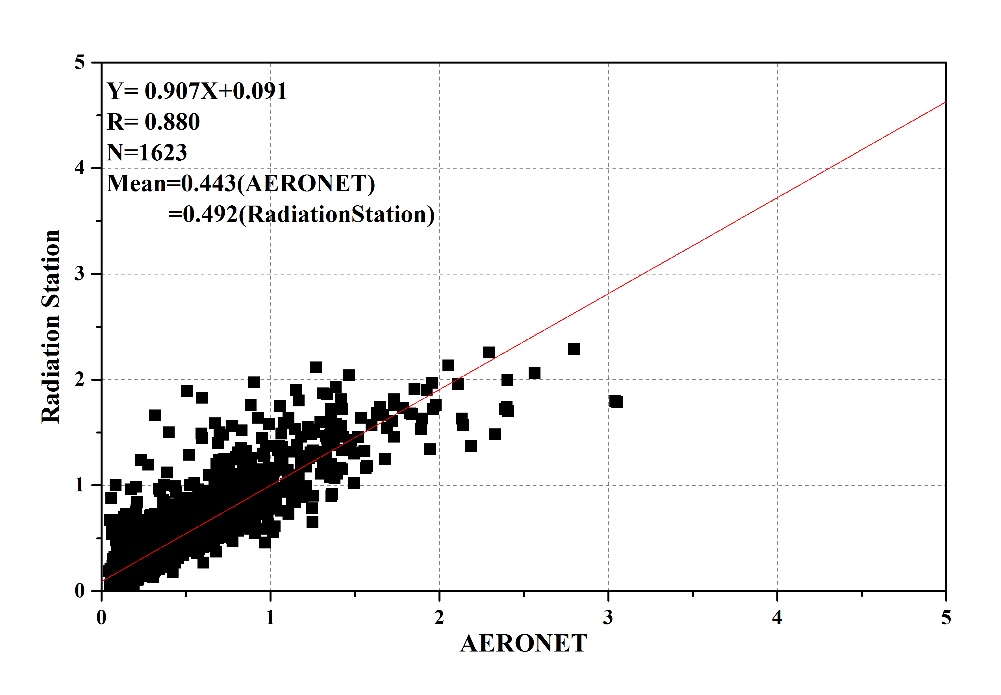


**Supplemental Figure S5 | The scatter diagram of daily radiation station retrieved AOD versus the AERONET observed AOD from 2001 to 2012 over Beijing.** The red line corresponds to the fitted line created by 1632 verification points.

| Table S1 \| Daily average meteorological parameters under different atmospheric conditions. | | | | |
| --- | --- | --- | --- | --- |
|  | HPBL(m) | RH(%) | TI_D(m) | TI_T(k) |
| Breezeless | 612.519 | 39.101 | 259.662 | 2.933 |
| South | 708.569 | 46.841 | 276.095 | 3.043 |
| North | 632.885 | 36.298 | 216.355 | 2.345 |
